# Comparison of two preclinical myocardial infarct models: coronary coil deployment versus surgical ligation

**DOI:** 10.1186/1479-5876-12-137

**Published:** 2014-05-21

**Authors:** Carolina Gálvez-Montón, Cristina Prat-Vidal, Idoia Díaz-Güemes, Verónica Crisóstomo, Carolina Soler-Botija, Santiago Roura, Aida Llucià-Valldeperas, Isaac Perea-Gil, Francisco M Sánchez-Margallo, Antoni Bayes-Genis

**Affiliations:** 1 ICREC (Heart Failure and Cardiac Regeneration) Research Program, IGTP, Cardiology Service, Hospital Universitari Germans Trias i Pujol, Crta. Can Ruti, Camí de les Escoles, s/n, 08916 Badalona, Barcelona, Spain; 2 Jesús Usón Minimally Invasive Surgery Centre, JUMISC, Cáceres, Spain; 3 Cardiology Service, Hospital Universitari Germans Trias i Pujol, Badalona, Spain; 4 Department of Medicine, UAB, Barcelona, Spain

**Keywords:** Myocardial infarction, Magnetic resonance imaging, Animal model surgery, Coil embolization, Surgical occlusion

## Abstract

**Background:**

Despite recent advances, myocardial infarction (MI) remains the leading cause of death worldwide. Pre-clinical animal models that closely mimic human MI are pivotal for a quick translation of research and swine have similarities in anatomy and physiology. Here, we compared coronary surgical ligation versus coil embolization MI models in swine.

**Methods:**

Fifteen animals were randomly distributed to undergo surgical ligation (n = 7) or coil embolization (n = 8). We evaluated infarct size, scar fibrosis, inflammation, myocardial vascularization, and cardiac function by magnetic resonance imaging (MRI).

**Results:**

Thirty-five days after MI, there were no differences between the models in infarct size (P = 0.53), left ventricular (LV) ejection fraction (P = 0.19), LV end systolic volume (P = 0.22), LV end diastolic volume (P = 0.84), and cardiac output (P = 0.89). Histologically, cardiac scars did not differ and the collagen content, collagen type I (I), collagen type III (III), and the I/III ratio were similar in both groups. Inflammation was assessed using specific anti-CD3 and anti-CD25 antibodies. There was similar activation of inflammation throughout the heart after coil embolization (P = 0.78); while, there were more activated lymphocytes in the infarcted myocardium in the surgical occlusion model (P = 0.02). Less myocardial vascularization in the infarction areas compared with the border and remote zones only in coil embolization animals was observed (P = 0.004 and P = 0.014, respectively).

**Conclusions:**

Our results support that surgical occlusion and coil embolization MI models generate similar infarct size, cardiac function impairment, and myocardial fibrosis; although, inflammation and myocardial vascularization levels were closer to those found in humans when coil embolization was performed.

## Background

Myocardial infarction (MI) is the leading cause of morbidity, mortality, and disability in the worldwide and remains one of the greatest challenges in biomedical research [1]. Current treatments including primary percutaneous coronary intervention, coronary artery bypass graft, or thrombolysis have significantly improved outcomes but do not restore myocardial damage after MI [2]. Consequently, new strategies such as cardiac cell therapy, tissue engineering, and gene therapy are being evaluated in pre-clinical models of disease [3]. Thus, for clinical translation, it is necessary to identify the best experimental model that mimics human MI conditions.

Numerous animal models evaluate the pathophysiological mechanisms of ventricular remodeling and MI development and progression [4,5]. Briefly, swine are mostly used because of their high similarity to humans, as they share minimal pre-existing coronary collaterals and have a similar coronary physiology and anatomy [6]. Despite acceleration of early myocardial healing process in swine compared to humans [7], histopathologic changes after MI are common with an earliest myocardial necrosis followed by infiltration of myofibroblast and inflammatory cells (i.e. neutrophils, macrophages, lymphocytes and plasma cells), and necrotic tissue replacement by collagen I and III [8]. MI pig model by permanent arterial occlusion can be induced following intracoronary ethanol administration [9], cryo-injury, cauterization [6], surgical ligation [10], and coil embolization [11]. Although ethanol, cryo-injury and cauterization are feasible and easily reproducible, these techniques promote pathophysiological changes different than those observed in humans [6]. Surgical coronary ligation causes irreversible damage to the myocardium and has been used extensively to assess cardiac regeneration following cell-based therapy and tissue engineering approaches. However, in contrast to human MI, the downside of this model involves an open-chest surgery. Alternatively, closed-chest options based on permanent coronary occlusion by coil deployment avoid the hassles of surgery, adjacent scarring, and post-operative inflammation.

The presented work aims to compare infarct size, histological traits and cardiac functional changes following surgical ligation or coil deployment. All interventions were performed in the first marginal branch of the circumflex coronary artery in swine and the follow-up period was 35 days.

## Methods

### Study design

Fifteen Landrace × Large White female pigs (20–30 kg) were randomly distributed into surgical coronary ligation (n = 7) and coronary coil deployment (n = 8) groups. All animals underwent MI induction in the first marginal branch of the circumflex coronary artery and were sacrificed after 35 days of follow-up. This study was approved by the local Animal Experimentation Unit Ethical Committee and complies with all guidelines concerning the use of animals in research and teaching as defined by the Guide for the Care and Use of Laboratory Animals (NIH Publication No. 80–23, revised 1996).

### Anaesthesia and analgesia

Pigs were premedicated as previously described [7]. In brief, propofol (4 mg/kg, IV; Recofol®, Bayer Schering Pharma) was used as an anaesthetic and, after endotracheal intubation, anaesthesia was maintained by 2% sevoflurane inhalation. A continued IV infusion of Ketorolac (0.15 mg/kg/h; Toradol®, Roche) and tramadol (0.5 mg/kg/h; Adolonta®, Grünenthal Pharma, S.A.) was used as an intra-operatory analgesia. At the beginning of the surgical procedures, a 1 mg/kg lidocaine bolus (IV, Lidocaína® 2%, B. Braun) was administered, followed by a continuous infusion (1 mg/kg/h) that was maintained for 1 h after MI induction. Finally, an IM dose of 2.5 mg/kg tulathromycin (Draxxin®, Pfizer Animal Health) was administered as a post-operative antibiotic. All surgery procedures were done under monitoring conditions with electrocardiogram (ECG) registration and measures of capnography, non-invasive arterial blood pressure, pulse oximetry, and temperature.

### Chronic MI models

#### Surgical coronary ligation

**Figure 1:**
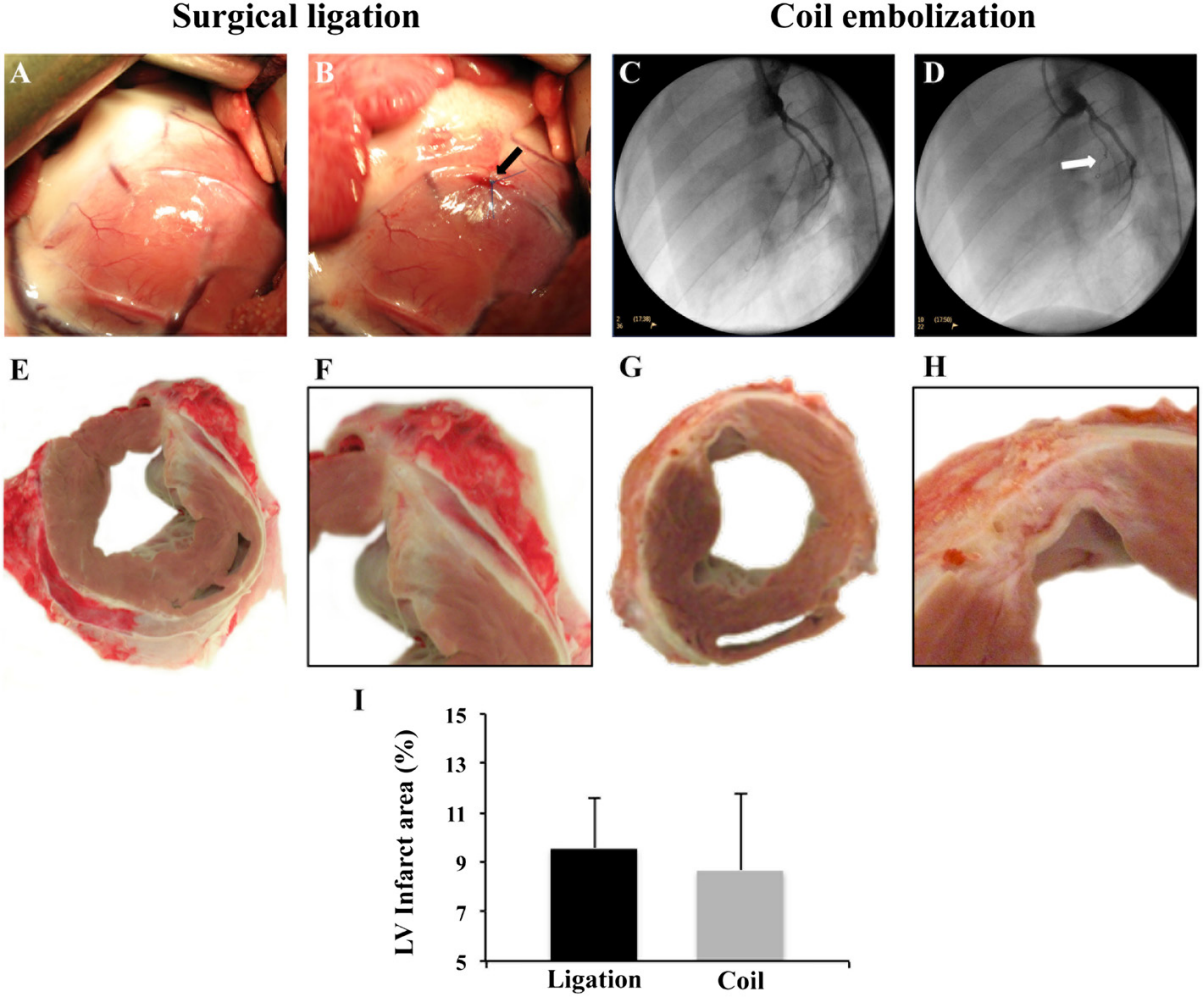
**Swine MI models (surgical ligation and coil embolization) and morphometric analysis.** The surgical coronary ligation model before **(A)** and after **(B)** arterial occlusion (arrow) with bruising of the affected tissue in purple. The coil embolization model before **(C)** and after **(D)** coil implantation under angiography, showing baseline coronary tree perfusion **(C)** and TIMI = 0 **(D)**. Representative images of the LV cardiac section showing MI after surgical occlusion **(E-F)** and coil deployment **(G-H)**. **(I)** Percentage of LV infarct area measured in hearts after 35 days of follow-up (P = 0.528).

MI was induced in 7 animals after coronary surgical ligation of the first marginal branch of the circumflex coronary artery (Figure 1A-B) as previously described [10]. Briefly, after lateral thoracotomy, MI was induced by a double-ligation (Prolene 5/0 W-8556 12-S, Ethicon Inc.) of the coronary artery, 1.5-cm distal from the atrioventricular groove The onset of ST segment elevation on the ECG and bruising of the affected tissue were used to assess MI.

#### Coil deployment

Eight animals underwent a coronary coil embolization in the first marginal branch of the circumflex coronary artery (Figure 1C-D) as previously described [11]. Briefly, after engaging the left main artery using a 6-F AR1 guide catheter, a left lateral MI was experimentally induced by percutaneous embolization coil deployment (VortX-18 Diamond 3 mm/3.3 mm coil, Boston Scientific/Target) in the first marginal branch of the circumflex artery under continuous fluoroscopy (BV Pulsera, Philips Medical Systems). In all cases, complete thrombotic occlusion (TIMI flow score = 0) was confirmed by coronary angiography 15 min after coil implantation.

### Cardiac function assessment

Cardiac function was evaluated by magnetic resonance imaging (MRI) performed at 1.5 T (Intera, Philips, Best, The Netherlands) using a phased-array cardiac coil [10,11]. All images were acquired under apnoea with continuous ECG gating. Left ventricular (LV) global function was assessed at baseline and at sacrifice, as previously described [6], and the ejection fraction (LVEF), end systolic volume (LVESV), end diastolic volume (LVEDV), and cardiac output (CO) were measured and compared between groups.

### Morphometric analysis

After premedication, pigs were sacrificed with a potassium chloride IV injection. LV infarct size was blindly measured in digitally photographed transverse heart sections from 1 cm distally to the coronary ligation or coil localization. Additionally, in order to confirm a correlation of MRI findings with gross pathology findings, LV infarct size was also analysed in gadolinium enhancement MRI images. Image-Pro Plus software (6.2.1 version; Media Cybernetics, Inc.) was used for quantitative morphometric measurements.

### Histological analysis

After sacrifice, hearts were excised and samples from the infarct core and remote myocardium were snap frozen. After 10% buffered formalin fixation, 5 μm tissue sections were stained using Masson’s and Gallego’s trichrome procedures for histological examination. In addition, Picrosirius red staining was performed to analyse myocardial fibrosis, as previously described [6]. The collagen volume fraction (CVF), collagen I, collagen III, and collagen I/III ratio were measured in the infarct core and remote zones with Image-Pro Plus software.

### Immunohistochemistry

In MI sections, inflammation was assessed by measuring the total anti-CD3 antibody positive lymphocytes, those labelled with anti-CD25 antibody, and the CD25/CD3 ratio. Briefly, sections were incubated with primary antibodies against CD3 (1:100; AbD Serotec) and CD25 (1:10; AbD Serotec) and with conjugated secondary antibodies using donkey anti-rat DyLight 488 (1:1000; Jackson ImmunoResearch) and donkey anti-mouse Cy3 (1:1000; Jackson ImmunoResearch), respectively. Vessel density in infarct, border, and remote zones was double-blindly assessed by Isolectin-B4 staining (1:25; Vectors Labs), Alexa Fluor 488-conjugated Streptavidin (1:500; Vectors Labs). Images were captured with a Leica TCS SP5 microscope (20x) and results were expressed as a percentage of the vessel area per field [8]. All sections were finally counterstained with Hoechst 33342 (1:10000; Sigma-Aldrich) and analysed with Image-Pro Plus software.

### Statistical analysis

Data are presented as the mean ± SD. All analyses were performed with SPSS statistic software (19.0.1 version, SPSS Inc.). Differences in morphometric and histological data between the surgical ligation and coil deployment groups were analysed using Student's t-test and cardiac function evolution was compared using ANOVA with the Greenhouse-Geisser correction. Statistical significance was achieved when P < 0.05.

## Results

None of the animals died during MI induction or follow-up and were sacrificed at the pre-defined time course (36 ± 1.5 surgical ligation vs. 37 ± 1.5 days coil embolization, respectively; P = 0.28).

### Infarct size

Morphometric analysis from digitally photographed transverse heart sections revealed that LV infarct sizes (Figure 1E-H) were similar when comparing surgical ligation and coil embolization groups at sacrifice (9.6 ± 2 vs. 8.7 ± 3.1%, respectively; P = 0.53) (Figure 1I). This result was also confirmed when LV infarct size was calculated in gadolinium enhancement MRI images (9.1 ± 6.6 vs. 11.6 ± 4.6%, respectively; P = 0.26).

### Cardiac function

**Figure 2:**
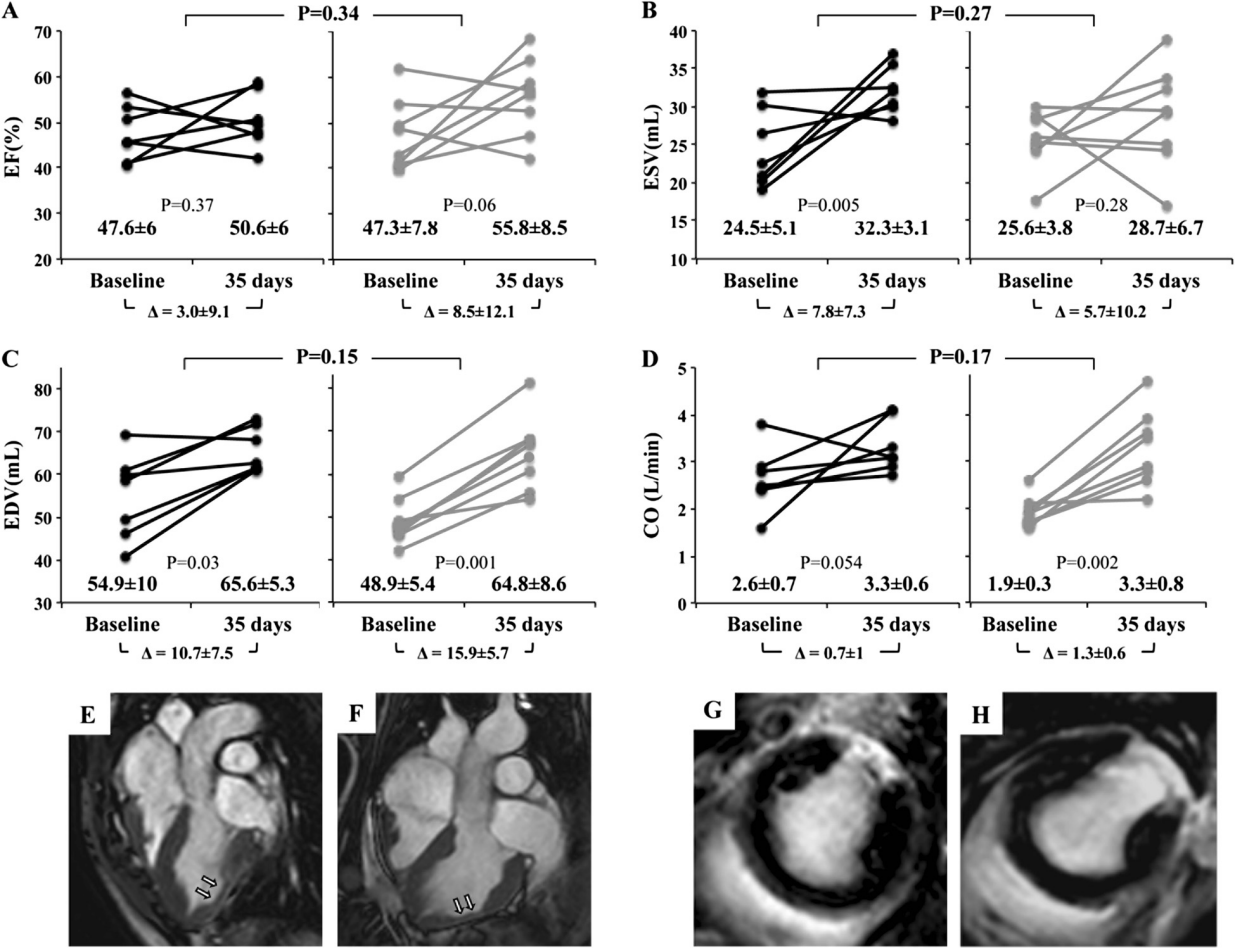
**Cardiac function analysis by MRI. (A-D)** LVEF, ESV, EDV, and CO at baseline and 35 days after MI in surgical occlusion and coil embolization animal models. Data for individual pigs (dots) and the mean (±SD) are shown. (P = 0.341, P = 0.269, P = 0.149, and P = 0.168, respectively). **(E and F)** Representative turbo-spin-echo and **(G and H)** T1 short-axis delayed enhancement images from the coronary ligation and coil deployment groups. Arrows indicate MI in the LV wall and T1 images show healthy myocardium (in black) and the MI with gadolinium retention (in white).

Baseline cardiac function was similar in both groups; in the surgical ligation versus coil embolization group with regards to LVEF (48 ± 6 vs. 47 ± 8%, respectively; P = 0.95), LVESV (25 ± 5 vs. 26 ± 4 mL, respectively; P = 0.62), and LVEDV (55 ± 10 vs. 49 ± 5 mL, respectively; P = 0.16). At sacrifice, ventricular function measurements were: LVEF (51 ± 6 vs. 56 ± 9%; P = 0.19), LVESV (32 ± 3 vs. 29 ± 7 mL; P = 0.22), and LVEDV (66 ± 5 vs. 65 ± 9 mL; P = 0.84). Although the measured baseline CO values were different between surgical and coil occlusion animals (2.6 ± 0.7 vs. 1.9 ± 0.3 L/min, respectively; P = 0.02), they did not differ at sacrifice (3.3 ± 0.6 vs. 3.3 ± 0.8 L/min, respectively; P = 0.89) (Online Table 1).To ensure a correct analysis of cardiac function, Greenhouse-Geisser analysis was applied to take into account the inter-individual differences in its evolution in each animal (baseline vs. sacrifice) when the two groups were compared. The results confirmed that there were no differences between groups in LVEF (P = 0.34), LVESV (P = 0.27), LVEDV (P = 0.15), and CO (P = 0.17) (Figure 2A-D). Two animals from each studied group were represented in Figure 2(E-H), which shows LV wall narrowing (E-F) and myocardial scar after gadolinium contrast injection (G-H).

**Table 1:** Cardiac function data

	**Surgical occlusion**	**Coil embolization**	***P *** **value**
**Baseline EF (%)**	47.6 ± 6.0	47.3 ± 7.8	*P* = 0.95
**Final EF (%)**	50.6 ± 6	55.8 ± 8.5	*P* = 0.195
**Baseline ESV (mL)**	24.46 ± 5.12	25.63 ± 3.85	*P* = 0.623
**Final ESV (mL)**	32.26 ± 3.11	28.68 ± 6.67	*P* = 0.217
**Baseline EDV (mL)**	54.88 ± 10	48.88 ± 5.41	*P* = 0.164
**Final EDV (mL)**	65.59 ± 5.28	64.82 ± 8.6	*P* = 0.84
**Baseline CO (L/min)**	2.62 ± 0.66	1.94 ± 0.32	*P* = 0.021
**Final CO (L/min)**	3.33 ± 0.56	3.27 ± 0.81	*P* = 0.885

Cardiac MRI data at baseline and 35 days post-MI (final) of pigs after surgical occlusion and coil embolization. Values represent the mean ± SD. EF indicates ejection fraction; ESV, end systolic volume; EDV, end diastolic volume; and CO, cardiac output.

### Histological and fibrotic changes

**Figure 3:**
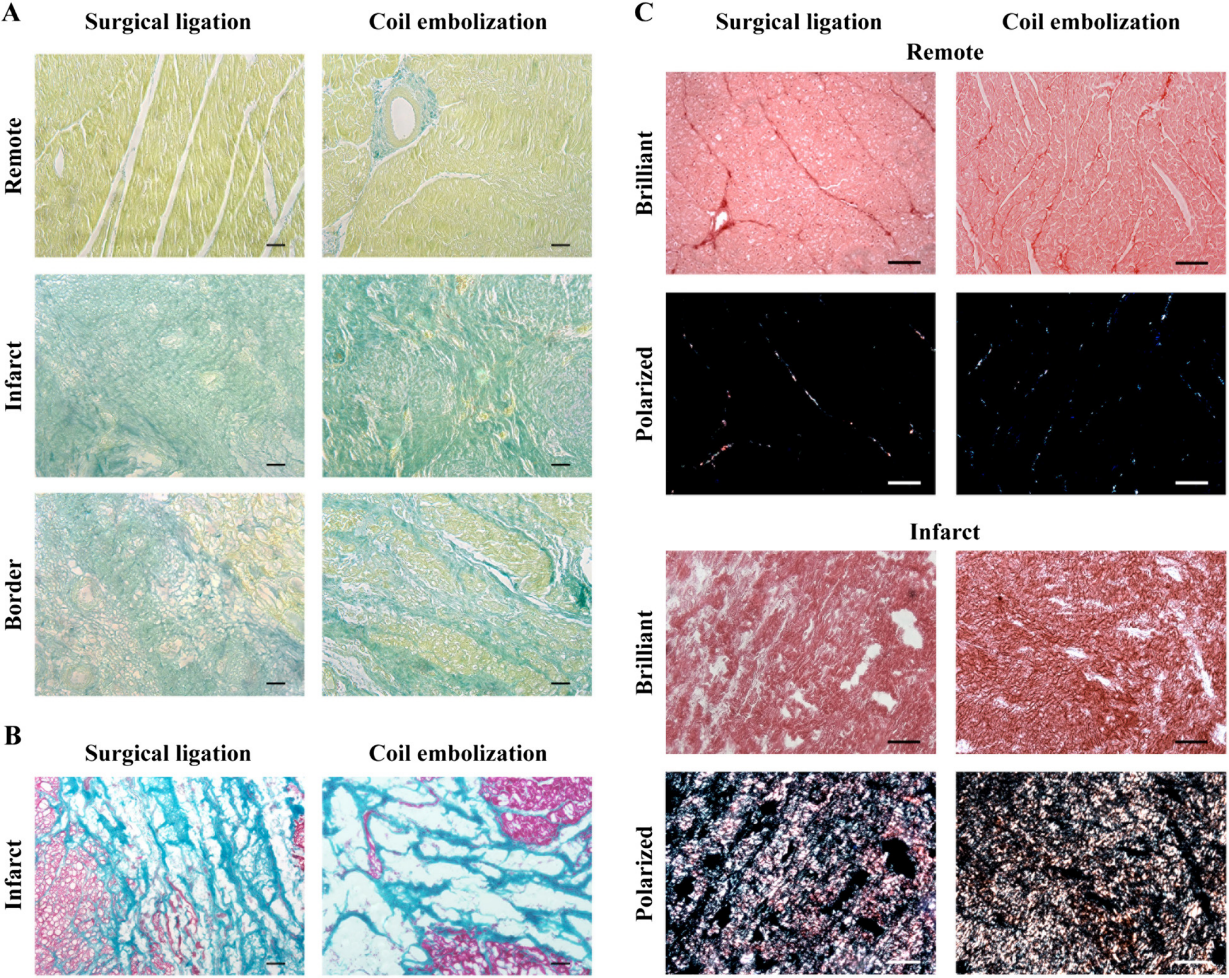
**Histological and myocardial fibrosis analyses. (A)** Gallego’s trichrome staining (scale bar = 100 μm) of remote, border, and infarct tissue sections showing collagen deposition (green), healthy muscle (yellow), and nuclei (pink). **(B)** Masson’s trichrome (scale bar = 100 μm) in MI heart sections, staining collagen scar in light green and healthy myocardium in Ponceau fuchsin. **(C)** Representative images of fibrosis analysis with Picrosirius red staining in remote and infarct myocardial zones under bright field, displaying collagen (red) and cardiac muscle (pink), and under polarized light showing collagen I (red/yellow) and collagen III (green) fibrils in the same tissue sections (scale bar = 50 μm).

Both Gallego’s (Figure 3A) and Masson’s (Figure 3B) trichrome staining demonstrated that the remote, border, and infarct zones of all animals were similar in the levels of fibrosis and necrotic tissue. Furthermore, there was no evidence of spontaneous regeneration at sacrifice in either group. Picrosirius red staining was used to analyse the fibrosis scar and confirmed there were no differences between the surgical occlusion and coil embolization groups in CVF (remote zone: 1.77 ± 0.78 vs. 1.83 ± 0.81%, respectively; P = 0.89) (infarct zone: 51.56 ± 14.74 vs. 37.31 ± 6%, respectively; P = 0.07), collagen I (remote zone: 0.78 ± 0.55 vs. 0.68 ± 0.34%, respectively; P = 0.68) (infarct zone: 33.35 ± 17.04 vs. 24.36 ± 13.35%, respectively; P = 0.35), collagen III (remote zone: 1 ± 0.39 vs. 1.15 ± 0.65%, respectively; P = 0.63) (infarct zone: 18.21 ± 5.94 vs. 12.94 ± 13.83%, respectively; P = 0.38), and the I/III ratio (remote zone: 0.80 ± 0.52 vs. 0.81 ± 0.68%, respectively; P = 0.97) (infarct zone: 2.35 ± 2.40 vs. 15.14 ± 21.57%, respectively; P = 0.26) (Figure 3C).

### Inflammation

**Figure 4:**
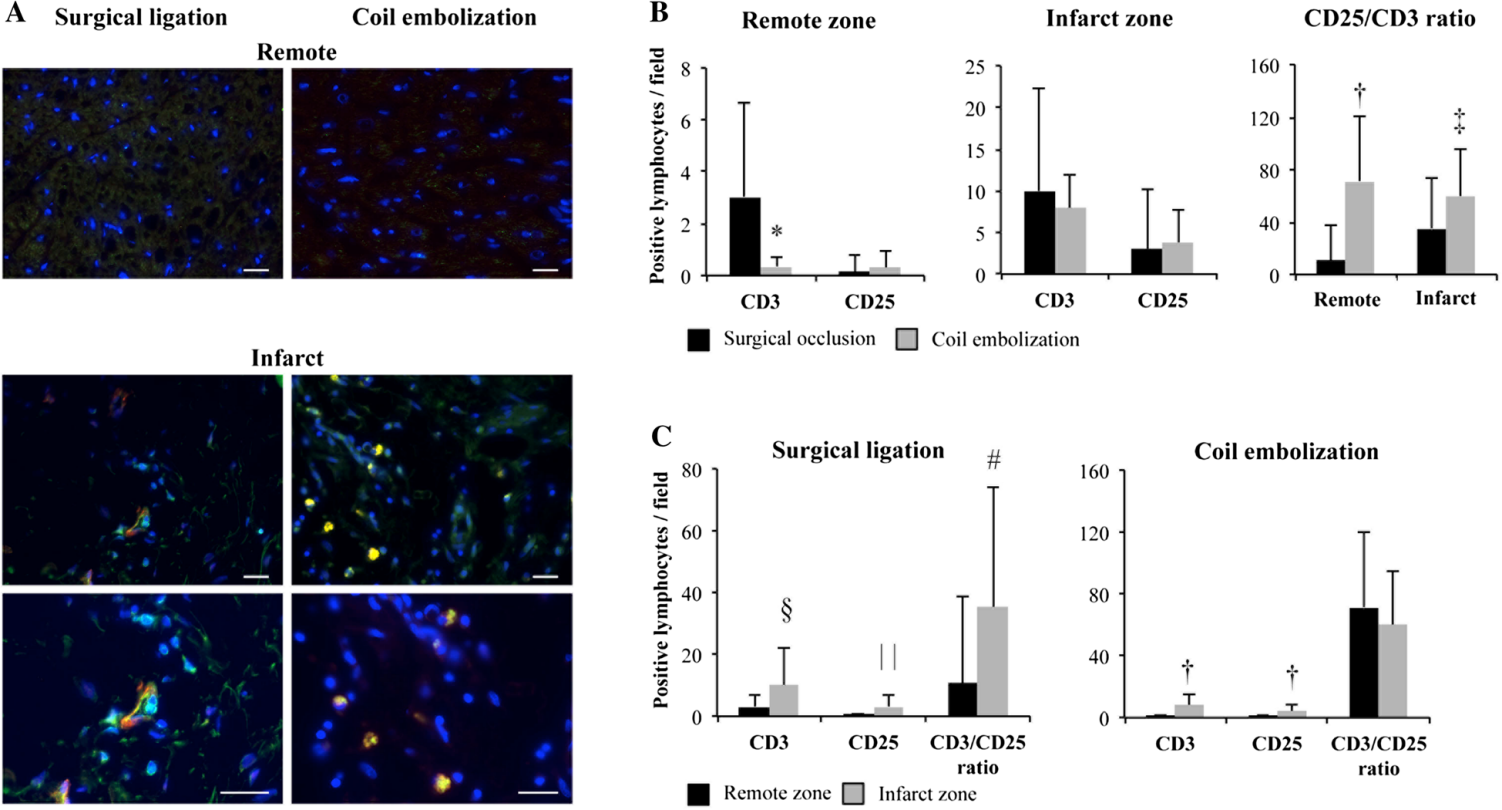
**Inflammation analysis. (A)** Immunohistofluorescence of remote and infarcted areas against CD3 (green) and CD25 (red) labeled lymphocytes and activated lymphocytes, respectively. Nuclei are counterstained with Hoechst 33342 in blue. At the bottom, zoomed images from the infarct core (scale bar = 50 μm). **(B)** Positive anti-CD3, CD25 lymphocytes and CD25/CD3 ratio per field in remote and infarct zones after surgical ligation vs. coil embolization (*P = 0.002, ^†^P < 0.001, and ^‡^P = 0.021). **(C)** Histograms comparing same inflammation data between remote and infarct zones in each animal model (^§^P = 0.008, ^| |^P = 0.001, and ^#^P = 0.023).

In contrast, inflammation analysis showed significant differences between the surgical occlusion and coil embolization procedures (Figure 4A) when CD3 (3.04 ± 3.59 vs. 0.35 ± 0.65 lymphocytes per field, respectively; P = 0.002) and the CD3/CD25 ratio (10.61 ± 27.9 vs. 83.33 ± 40.82% of activated lymphocytes, respectively; P < 0.001) were measured in the remote zone and the CD3/CD25 ratio (33.37 ± 38.82 vs. 59.94 ± 35.36% of activated lymphocytes, respectively; P = 0.021) was measured in the infarct zone (Figure 4B). Moreover, when remote and infarct zones were compared in each group, there were differences in CD3 (3.04 ± 3.59 vs. 10 ± 12.33 lymphocytes per field, respectively; P = 0.008), CD25 (0.13 ± 0.34 vs. 3.07 ± 3.95 activated lymphocytes per field, respectively; P = 0.001), and the CD3/CD25 ratio (10.61 ± 27.9 vs. 35.37 ± 38.82% of activated lymphocytes, respectively; P = 0.023) in the surgical occlusion group and in CD3 (0.35 ± 0.65 vs. 7.96 ± 7.24 lymphocytes per field, respectively; P < 0.001) and CD25 (0.30 ± 0.63 vs. 3.82 ± 3.95 activated lymphocytes per field, respectively; P < 0.001) in the coil embolization group (Figure 4C). In the coil embolization group, the ratio did not differ between the infarct and remote zones, which showed similar activation of the inflammation process throughout the heart (P = 0.78) whereas the activity of lymphocytes was increased in the infarcted myocardium in the surgical occlusion group.

### Vascularisation

**Figure 5:**
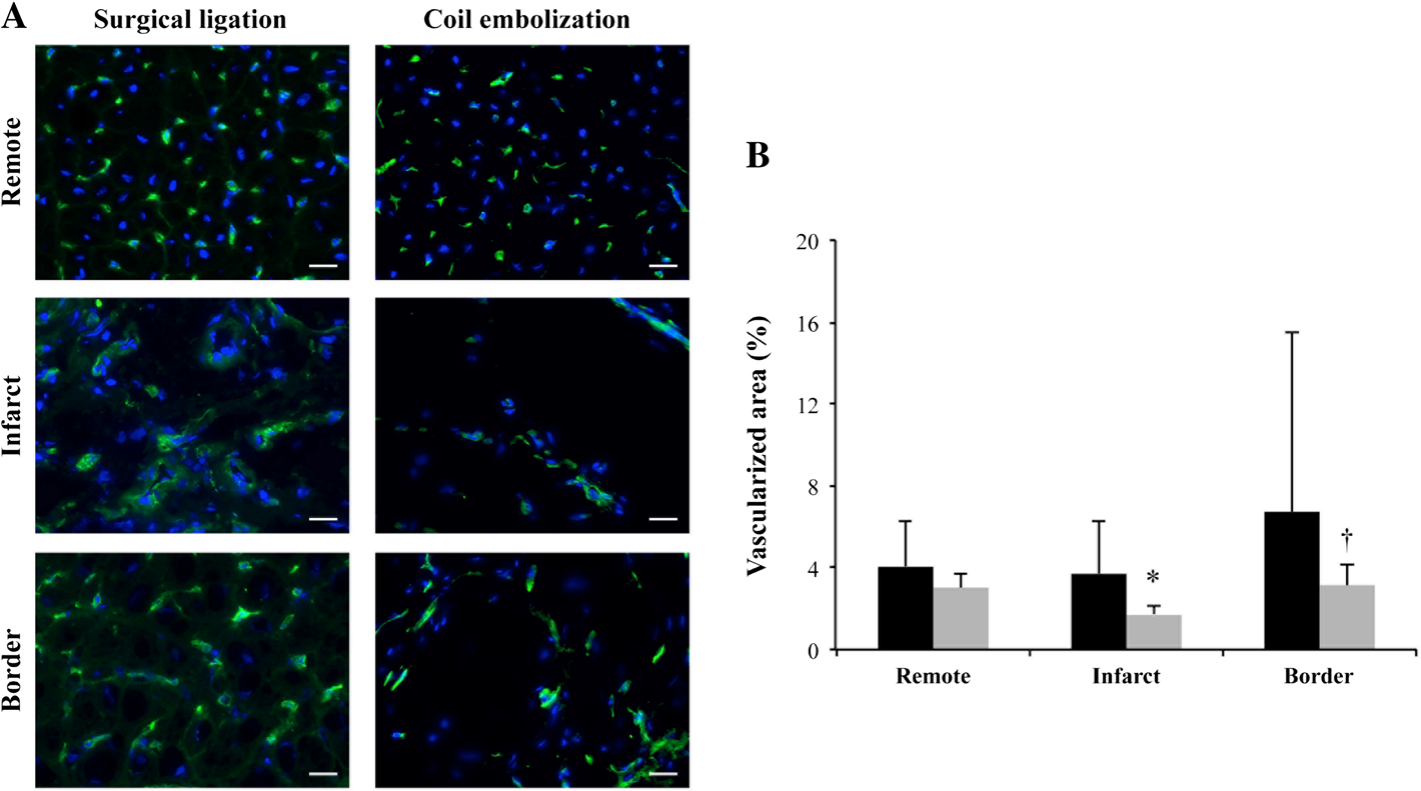
**Vascularization analysis. (A)** Representative immunohistofluorescence images of Isolectin-B4 (green) and nuclei-Hoechst 33342 (blue) staining showing significant differences in the infarct zone versus remote and border myocardium in the coil embolization model (scale bar = 50 μm). **(B)** Percentage of vascularized area in the same myocardial zones (*P = 0.014 and ^†^P = 0.004).

After Isolectin B4 staining, no differences in the vascularized area between the surgical ligation and coil deployment groups in remote (4.08 ± 2.44 vs. 3.02 ± 0.75%, respectively; P = 0.35), infarct (3.73 ± 2.79 vs. 1.74 ± 0.35%, respectively; P = 0.14), and border (6.76 ± 9.62 vs. 3.14 ± 1%, respectively; P = 0.4) zones were detected. Nevertheless, coil deployment animals showed significant differences in the vascularized areas when comparing the infarct zone with remote (P = 0.014) and border (P = 0.004) myocardium. All zones appeared similar in the surgical ligation group animals (P = 0.99; P = 0.66; respectively) (Figure 5 and Additional file 1: Figure S1).

## Discussion

The results of this study confirm that MI, either by surgical occlusion or coil deployment, is similar in terms of infarct size, cardiac function impairment, and myocardial fibrosis, but presents significant differences in myocardial vascularization and inflammation properties when the two techniques are compared.

Currently, MI experimental studies are crucial for testing efficiency of cardiac cell therapy and tissue engineering and ultimately for translational medicine. In this field of research, swine are extremely well suited animals. Several MI models have been suggested for surgical approaches and percutaneous techniques. Although surgical models allow direct visual MI assessment, are feasible, and presume to be easily reproducible, they have an undesired mortality rate (10-50%) and involve open-chest surgery [12]. Moreover, an open-chest approach increases the risk of infections [13] and comprises postoperative reactive inflammation as well as the establishment of new epicardial collateral vessels [14]. Collectively, these represent substantial differences in comparison with human MI. In the present work a coronary occlusion on the marginal branch of the left circumflex artery was performed to reduce mortality caused by MI induction and evolution. Indeed, in our hands, there was no evidence of arrhythmias or lethal events recorded in these animal series.

Cardiac MRI has become the gold-standard for assessing ventricular function due to its high reproducibility; in addition, this procedure requires fewer animals to evaluate a hypothesis regarding adverse remodeling data [15]. In the present study, MRI analysis demonstrated that global cardiac functional parameters such as LVEF, LVESV, LVEDV and CO did not differ between the two groups 35 days after MI induction. However, LVEF and cardiac output have appeared higher after the MI than at baseline. This result may be partly explained by the juvenile swine model which is still growing with immature cardiac function; by the limited infarct size caused by occluding the first marginal branch of the circumflex coronary artery; and also eventually by cardiac remodelling and neurohormonal activation driven by the cardiac injury. Although there was no LVEF impairment, all animals suffered an adverse remodeling process after MI induction where ventricular volumes significantly increased at sacrifice. It is well known that LVESV is an important parameter to evaluate heart failure progression after MI and, consequently, is directly responsible for the remodeling phenomena [16,17]. Moreover, myocardial remodeling also includes cell apoptosis, necrosis, and alteration of the balance between myocardial extracellular matrix and collagen fiber synthesis and degradation [18]. In this study, myocardial fibrosis was present after MI and was similar in all animals with comparable CVF values and collagen I/III ratios in the infarcted zone.

On the other hand, the inflammatory response after MI is crucial for myocardial healing and repair, diminishing tissue injury and regulating scar formation [19]. The inflammatory process is characterized by an early macrophage and neutrophil infiltration, specific complement activation, secretion of cytokines and, lastly, increasing levels of T and B cells [20]. Notably, Varda-Bloom *et al.* also described a slight mononuclear inflammation in the remote areas of infarcted hearts, finding lymphocytes between healthy muscle fibers. Our findings confirm this presence both in coronary surgical ligation and coil embolization models. In particular, the level of CD3+ cells in the remote myocardium was notably increased after open-chest model although not so activated as in coil embolization model. In addition, lymphocyte activation (normalized to the total number of CD3^+^ cells) was also increased in the infarcted area of coil animals. Thus, lymphocyte infiltration in the whole heart after MI is more evident after coronary surgical ligation, probably due to the open-chest approach, as Li *et al*. suggested [13]. However, further long-term studies are needed to evaluate whether lymphocyte activation remains preserved over time leading to deleterious consequences. Furthermore, the higher lymphocyte activation after coil embolization could be explained by the chronic inflammation reaction at the interface between coil and tissue [21]. Hence, the findings presented here support to a better understanding of myocardial inflammation and remodeling processes after MI and also provide new key information to determine the correct MI model.

Interestingly, the growth of subendocardial collateral vessels after acute coronary occlusion has been described both in pigs [22] and humans [23], and was reported as a critical mechanism in the cardiac tissue salvage after injury. As mentioned above, the newly formed neo-vessels after the open-chest approach [11] could also improve myocardial healing, differing from what occurs in the natural human MI process, Although Isolectin B4 staining does not distinguish newly formed vessels, this assumption is suggested from our results due the vascular similarities found between the infarct and border zones with remote healthy myocardium after surgical coronary ligation. On the contrary, myocardial vascularization after MI was significantly diminished in infarcted and border zones in animals operated on the closed-chest approach, as in humans. Although these differences do not alter the infarct size or ventricular function, they should be considered when conducting studies of vascularization after MI. Consequently, coronary surgical occlusion may not be the most representative of clinical coronary ischemic events and alternatives closely resembling pathophysiological traits in humans should be used. Closed-chest percutaneous techniques, which avoid the new collateral development that occurs with open-chest procedures, appear to be a good option, including permanent balloon occlusion and embolization coils [24]. Balloon coronary angioplasty triggers frequent ventricular fibrillation with high mortality rates and its use is better suited in ischemia/reperfusion rather than permanent occlusion studies [12]. In contrast, coil embolization can be applied at any point of the coronary tree to model acute and chronic MI, or chronic congestive heart failure [18]. This alternative appears to be an excellent choice for cell-based therapy, cardiac tissue engineering, gene therapy, and pharmacologic studies in MI in comparison to open-chest coronary occlusion that could interfere with results and may hamper translation to humans.

## Conclusion

In summary, our results support that surgical occlusion and coil embolization MI models generate similar infarct size, cardiac function impairment, and myocardial fibrosis in swine. However, both inflammation and myocardial vascularization levels were closer to those found in humans when coil embolization was performed. Therefore we suggest the use of coil embolization in pre-clinical MI research, being a better MI model than surgical coronary ligation, to obtain reliable results and leading to prompt clinical applications.

### Limitations of the study

Landrace × Large White prepuberal pigs were used and, thus, results may differ from an adult model. For this purpose, minipigs represent a more regular alternative although they require more complex and expensive handling. Moreover, the ischemic model used in this study produces a limited infarct size, without the induction of malignant ventricular arrhythmias and showing limited effects on ventricular function. In addition, the lack of echocardiographic monitoring prevents the evaluation of ischemic mitral valve regurgitation, frequently observed in lateral LV infarction.

## Abbreviations

MI: Myocardial infarction; ECG: Electrocardiogram; MRI: Magnetic resonance imaging; LV: Left ventricular; LVEF: Left ventricular ejection fraction; LVESV: Left ventricular end systolic volume; LVEDV: Left ventricular end diastolic volume; CO: Cardiac output; CVF: Collagen volume fraction.

## Competing interests

The authors declare that they have no competing interests.

## Authors’ contributions

CGM carried out the animal experimental set, participated in its design, performed the statistical analysis, and drafted the manuscript. CPV and IDG helped to carry out the experimental procedures. VC carried out MRI analysis. CSB, SR, ALV and IPG carried out the histological and immunohistochemical analysis and helped in acquisition data. FSM and ABG participated in its design and drafted the manuscript. All authors read and approved the final manuscript.

## Supplementary Material

Additional file 1: Figure S1 Vascularization analysis by Gallego’s trichrome staining. Representative images showing vascular structures (arrows) in myocardial sections including infarct, border and remote zones after surgical occlusion and coil embolization corroborating Isolectin B4 staining data. Note that Gallego’s trichrome technique clearly distinguishes blood vessels (yellowish green) from collagen (dark blue), muscle fibers (yellow), and nuclei (pink). (Scale bar = 100 μm). Click here for file
